# American Society of Dental Surgeons

**Published:** 1844-09

**Authors:** 


### Miscellaneous Notices.

### AMERICAN SOCIETY OF DENTAL SURGEONS.

The Fifth Annual Meeting of this Association was held in the Hall of the Medical Department of the university of New York, and we were gratified to perceive among the members in attendance, as it gave evidence of unabated zeal for the advancement of the objects of the Society, individuals from distant and remote parts of the Union. The meeting was more numerously attended than any of the previous ones of this body; and throughout the whole of its deliberations the utmost harmony and good feeling prevailed; all seemed actuated by one common object, namely, the elevation of the standard of professional qualification, the suppression of empiricism, and the diffusion of a correct knowledge of the theory and practice of Dental Surgery.

The business of the Society, although very considerable, was gone through with in two days, and much praise is due to our newly elected President, Dr. E. Parmly, for the able and dignified manner in which he presided. A better presiding officer could not have been chosen, for while he commands respect, he at the same time secures the esteem and friendship of all.

The names of the members whom we recollect to have seen in attendance upon the meeting are, Drs. E. Parmly, J. Parmly, O. Holmes, L. Parmly, S. M. Shepherd, J. W. Shepherd, John A. Cleaveland, E. Townsand, E. Laroque, A. Westcott, Ed. Maynard, J. Alcock, J. B. Rich, E. J. Dunning, C. Walker, G. G. Brewster, C. Meritt, H. Porter, S. Brown, A. W. Brown, W. G. Lord, C. C. Allen, J. H. Foster, J. M. Peak, B. Lord, E. Bryan, C. S. Rowell, M. K. Bridges, G. Merryman, J. Lovejoy, A. Hill, S. W. Parmly, S. W. Stockton, N. Dodge, A. C. Hawes, F. H. Clark, E. Blake, W.H. Dwinelle, P. Smith, — Cone, — Palmer, —Fuller, — Keese, and C.L. Norton. There were others present, whose names we cannot now call to mind.

The members of the Society having been notified of the place of meeting, assembled at the Hall of the Medical Department of the University of New York, and, at 11 o'clock, Dr. Eleazar Parmly, first Vice-president, took the chair; the minutes of the preceding meeting were then read and approved. After which, Dr. C. A. Harris rose, and stated that he did so, for the performance of a melancholy and painful duty; it was to announce the decease of one of the most prominent members of this body. Since our last meeting, said he, death has been abroad in our ranks, and has stricken down one whose name is dear to science, and who will long be remembered as the one who originated and assisted in the organization of this Society.

Dr. Hayden, continued he, our late venerable President, is no more. Dr. H. briefly alluded to his contributions to Dental Surgery, and to other departments of science; he stated that he had intended to have adverted to some of the most prominent incidents of his long and useful life; but having been anticipated in the remarks which he had intended to have offered, by his colleague, Professor Bond, in a valedictory delivered by him before the graduates of the Baltimore College of Dental Surgery, at the last Annual Commencement of that Institution, and which had subsequently been published in the American Journal of Dental Science, he should content himself with merely moving the appointment of a committee to draft resolutions expressive of the feelings of this body in relation to his demise.

The resolution was unanimously adopted, and Drs. Harris, Maynard, Brown, and E. Parmly, were appointed that committee.

The next business of the Society was the election of a President, to fill the vacancy occasioned by the death of Dr. Hayden, and Dr. Eleazar Parmly having been put in nomination by Dr. Harris, was ballotted for and unanimously elected.

Dr. Parmly returned thanks to the Society, for the united confidence which they had reposed in him, and offered some feeling remarks in relation to the decease of our late venerable President, who had been the head of the Society in its earliest formation, and its advocate and projector. He alluded to the lessons of instruction which our departed President had given us, and expressed his most ardent hope that we might profit by them, and thus transmit this institution to our successors, in that integrity and purity which its projector had so ardently desired, for which he had done so much, and written and spoken so wisely.

Dr. Parmly having concluded his remarks, Drs. Harris, Westcott and Townsand were appointed a committee to arrange the order of the future business of the meeting.

The above committee, after having retired, soon returned and made their report, the details of which, however, we do not think it necessary to give, as we propose to notice only such of the proceedings of the Society as would be likely to be of interest to the readers of the Journal.

One of the most important acts of the Society at its last meeting, was the revision and alteration of its Constitution and By-Laws, so as more effectually to guard against the admission to membership of unworthy individuals. And that the objects of the association and rules by which it is governed may be fully known and understood, we again publish entire, in another part of the Journal, its Constitution and By-Laws, as revised and amended.

At 2 o'clock, P. M., the Society adjourned to meet again at 4, when Dr. S. Brown delivered, extemporaneously, the opening address, which was attentively listened to by the members. The address was highly interesting, and appropriate to the occasion; it, however, excited general regret, as the

Dr., in the course of his remarks, announced his intention to retire from the active duties of his profession. He thanked the Society, in very feeling terms, for the continued confidence it had reposed in him, for its kindness in conferring upon him official station, and declined for the future any other relationship to it than that of a private member.

After Dr. B. had concluded, Dr. E. Parmly rose and made some very appropriate remarks in response to his address, alluding, in terms of the warmest praise, to the valuable services which he had rendered the Society, not only in its formation, but also during the whole period of its existence, up to the present time. In expressing his own regret at losing so valuable a coadjutor, he doubted not, that he expressed the feelings of every member of the Society.

When Dr. Parmly took his seat, Dr. Harris offered the following resolutions, which were unanimously passed:

*Resolved,* That it is with sincere and deep regret that we receive the announcement of the intention of our highly esteemed friend and brother, Dr. S. Brown, to retire from the active duties of the profession, and that the thanks of the Society be tendered him, for the great and important services he has rendered it, not only at the time of its organization, but also in its subsequent operations; and,

*Resolved, furthermore,* That he be requested to furnish a copy of the highly interesting address just delivered by him, for publication in the Journal.

At 6 o'clock, the Society adjourned to meet at half past 8, at Dr. E. Parmly's residence, No. 1 Bond street, which, when it had convened, it proceeded to the election of its officers for the next ensuing twelve months, which are as follows, including the President, who had been elected in the morning:

| | |
|---|---|
| ELEAZAR PARMLY, M. D. | PRESIDENT. |
| N. C. KEEP, M. D. | 1ST VICE-PRESIDENT. |
| E. TOWNSAND, D. D. S. | 2D VICE-PRESIDENT. |
| JOHN A. CLEAVELAND, D.D.S. | 3D VICE-PRESIDENT. |
| CHAPIN A. HARRIS, M. D | CORRESPONDING SECRETARY. |
| AMOS WESTCOTT, M. D. | RECORDING SECRETARY. |
| CHAPIN A. HARRIS, M. D. | TREASURER. |
| J. H. FOSTER, M. D. | LIBRARIAN. |

*Editors of the American Journal and Library of Dental Science.*

CHAPIN A. HARRIS, M. D. EDWARD MAYNARD, M. D.

AMOS WESTCOTT, M. D.

*Executive and Examining Committee.*

EDWARD MAYNARD, M. D. OLIVER HOLMES, D. D. S.

EDWARD LAROQUE. M. K. BRIDGES, D. D. S.

J. M. PEAK, M. D. S. M. SHEPHERD, D. D. S.

LEONARD MACKALL, M. D.

*Publishing Committee.*

LEWIS ROPER, M. D. E. TOWNSAND, D. D. S.
H. S. BURR, M. D.

J. H. Foster, M. D. was appointed to deliver the opening address, and the following gentlemen to deliver dissertations at the next meeting of the Society; namely, E. Townsand, D. D.S., E. J. Dunning, John Harris, M. D., J. M. Peak, M. D., and James Taylor, M. D.

The Society, after having transacted some other business, adjourned to meet the next (Wednesday) morning, at 9 o'clock, at the Hall of the Medical Department of the University of New York.

The first business which engaged the attention of the Society, after it met, Wednesday morning, was the report of Drs. Maynard, Holmes, Townsand, Harris, Cleaveland, and E. Parmly, the committee to whom had been referred the revision and alteration of the Constitution and By-Laws; so much of the action of the committee as related to the admission of members having been adopted, the further consideration was deferred until evening.

Among the applicants for membership, the following were admitted; Jesse W. Cook, M. D., Cincinnati, O.; T. P. Cleaveland, Augusta, Ga.: R. H. Harrison, M. D., Huntsville, Ala.; Dr. A. A. Cary, Huntsville, Ala.; Dr. Henry Crane, Cincinnati, O.; Wm. Bellangee, Montgomery, Ala.; Dr. Reynolds, Columbia, S. C.; Moses Holmes, Baltimore; Charles Bonsell, Cincinnati, O.; W. P. Smith, Binghampton, N. Y.; Dr. Bardwell, Troy, N. Y.; John W. Spear, Augusta, Ga.; Dr. M. Bissell, Charleston, S. C.; C. L. Norton, N. Y.; N. S. Tyler, Providence, R. I.; W. F. Hollyman, Columbia, S. C., Ed. Shuman, Paris, Ky.; Dr. Miller, Worcester, Mass.; Dr. Palmer, Poughkeepsie, N. Y.; H. F. Smith, White Plains, N. Y.; — Cone, Mass.; Ed. Jamet, Baltimore; E. D. Crispen, Philadelphia; — Keese, Danbury, Conn.; — Fuller. Part of the above were subjected to a rigid examination previously to their election.

After transacting some other business, among which was the adoption of a resolution instructing the Treasurer to send to members in arrears to the Society, a statement of their indebtedness, requesting immediate payment of the same, the following resolution was passed, namely,

***Resolved,*** That the Recording Secretary be instructed to give written notice to every individual of this Society, charged by another member with plugging teeth with *mineral paste* of *any* kind, that the Society has pronounced the use of that class of articles for this purpose *malpractice,* and such member, thus charged, that this Society will act upon his case at its next meeting.

The hour of 2 having arrived, the Society adjourned until 4, P. M., when Dr. Westcott delivered a dissertation on the "Claims of Medical Science" upon the Dental Surgeon, which was requested by the Society for publication; and to this able and highly interesting paper we invite special attention. We hope every reader of the Journal will give it a thorough and careful perusal.

After Dr. Westcott had concluded, Dr. O. Holmes offered two resolutions, which were adopted ; but as they were subsequently incorporated into the Constitution of the Society, it is not necessary to give them here.

The propriety of the formation of State Societies, auxiliary to this, was discussed at considerable length, by Drs. S. M. Shepherd, Jahial Parmly, A. Westcott, G. Merryman, O. Holmes, Ed. Maynard, C. A. Harris and others. Resolutions were also offered for the appointment of a committee to report a plan for the accomplishment of this object, at the next meeting of the Society, but having to meet a committee, just at this stage of the proceedings, we do not know whether any of them were adopted or not. Dr. Peak, however, afterwards offered a resolution, which was passed, recommending the formation of State Societies, by the members of this, in the states in which they respectively reside, not only for the purpose of accomplishing local good, but also for that of ultimately becoming auxiliary to the American Society of Dental Surgeons.

Dr. Alcock having kindly invited the members of the Society to an entertainment at his house in the evening, it was determined to transact the remainder of the business there; but before the meeting of the Society at the Hall of the University had adjourned, the following resolution was passed:

*Resolved,* That the thanks of the American Society of Dental Surgeons be tendered, by Dr. Eleazar Parmly, its President, to the Trustees of the Medical Department of the University of New York, for the very liberal tender of the College for the use of this Society, during its present Annual Session.

The Society adjourned its Wednesday afternoon session at half past 6 o'clock, and met at 8, at Dr. Alcock's, in Murray street. The Constitution and By-Laws, as altered and amended, were now read and adopted, article by article, and section by section.

After having been gone through with, Drs. O. Holmes and Merryman were appointed a committee to superintend the printing of them, with instructions to have five hundred copies published in cheap pamphlet form, for distribution among the members.

Resolutions expressive of the feelings of the Society, upon the occasion of the announcement of the demise of its late President:

*Resolved,* That the American Society of Dental Surgeons have heard with heartfelt sorrow, of the demise, since our last meeting, of the venerable, distinguished, and beloved President of this Sociery, Dr. Horace H. Hayden, of Baltimore.

*Resolved,* That this Society entertain an exalted opinion of the justice and dignity with which its late lamented President presided over its deliberations and actions, during its organization, and up to the time of his death.

*Resolved,* That, as a token of the great regard in which we hold the memory of our late President, for his services to mankind, and officially to this Society—for his many virtues, displayed in all the duties of his life—the members of this Society will wear the official badge of mourning thirty days.

*Resolved,* That a copy of these resolutions be sent to the family of our late President, with the assurance of profound grief for the irreparable loss which they and this Society have mutually sustained.

The Society now adjourned to the first Tuesday in August, 1845.—*Balt. Ed.*

